# A spatially comprehensive, hydrometeorological data set for Mexico, the U.S., and Southern Canada 1950–2013

**DOI:** 10.1038/sdata.2015.42

**Published:** 2015-08-18

**Authors:** Ben Livneh, Theodore J. Bohn, David W. Pierce, Francisco Munoz-Arriola, Bart Nijssen, Russell Vose, Daniel R. Cayan, Levi Brekke

**Affiliations:** 1 Cooperative Institute for Research in Environmental Science (CIRES), University of Colorado, Boulder, 216 UCB, Boulder, Colorado 80309, USA; 2 NOAA Earth System Research Laboratory Physical Sciences Division, 325 Broadway, Boulder, Colorado 80309, USA; 3 School of Earth and Space Exploration, Arizona State University, Tempe, AZ, 781 E. Terrace Mall, Tempe, Arizona 85287-6004, USA; 4 Climate, Atmospheric Sciences, and Physical Oceanography, Scripps Institution of Oceanography, La Jolla, California 92093-0224, USA; 5 Department of Biological Systems Engineering, University of Nebraska-Lincoln, 246L. W. Chase Hall P. O. Box 830726, Lincoln, Nebraska 68583-0726, USA; 6 Department of Civil and Environmental Engineering, University of Washington, Box 352700, Seattle, Washington 98195, USA; 7 NOAA/National Climatic Data Center, 151 Patton Avenue, Asheville, North Carolina 28801, USA; 8 Water and Climate Research Coordinator, U.S. Bureau of Reclamation

**Keywords:** Water resources, Hydrology, Environmental sciences

## Abstract

A data set of observed daily precipitation, maximum and minimum temperature, gridded to a 1/16° (~6 km) resolution, is described that spans the entire country of Mexico, the conterminous U.S. (CONUS), and regions of Canada south of 53° N for the period 1950–2013. The dataset improves previous products in spatial extent, orographic precipitation adjustment over Mexico and parts of Canada, and reduction of transboundary discontinuities. The impacts of adjusting gridded precipitation for orographic effects are quantified by scaling precipitation to an elevation-aware 1981–2010 precipitation climatology in Mexico and Canada. Differences are evaluated in terms of total precipitation as well as by hydrologic quantities simulated with a land surface model. Overall, orographic correction impacts total precipitation by up to 50% in mountainous regions outside CONUS. Hydrologic fluxes show sensitivities of similar magnitude, with discharge more sensitive than evapotranspiration and soil moisture. Because of the consistent gridding methodology, the current product reduces transboundary discontinuities as compared with a commonly used reanalysis product, making it suitable for estimating large-scale hydrometeorologic phenomena.

## Background & Summary

Observation-based meteorological data sets offer insights into changes to the hydro-climatic system by diagnosing spatio-temporal characteristics^1^ and providing a historical baseline for future projections^2^. Spatially consistent transboundary hydroclimatic data have the potential to reduce cross-border water disagreements by enabling equitable allocation and access to water resources^3^. Yet, creating multi-decadal data sets that span international boundaries presents a unique challenge, specifically to avoid discontinuities and artifacts related to data availability, collection protocols, and quality. Such artifacts can subsequently impact key transboundary water-related estimates, such as fish stocks^4^, water sharing agreements^5^, historical hydroclimatic assessments^6^, hydrologic forecasting^7,8^, and regional hydroclimatic phenomena such as the North American Monsoon (NAM^9–11^). Here, we applied a previously published methodology^12^ to create a sub continental daily data set of gridded station precipitation and maximum and minimum temperatures with minimal transboundary discontinuity.

Although remote sensing products offer estimates of other hydro-climatic quantities without transboundary discontinuities, e.g., evapotranspiration (ET), terrestrial water, and soil moisture, such estimates are limited in their potential to close the land surface water budget^13,14^, may be unconstrained by available water^15^, and generally have temporal frequencies that may be too coarse for daily applications^16,17^. While satellite precipitation products are available at daily or finer timescales, they tend to have large uncertainties^18^ and lack record lengths of more than 20 years needed to assess low recurrence phenomena such as extreme hydrometeorological events and the effects of ocean-atmosphere oscillations.

Precipitation and near-surface temperature records can be used to drive land surface models that explicitly close the water balance^19^. For example^20,21^ global gridded observations exist to this end, but at coarse spatial resolution (0.5°). High-resolution datasets are available from re-analyses (0.125° for NLDAS2^22^), although transboundary or temporal discontinuities emerge through incorporation of satellite data (0.3° for NCEP/NCAR reanalysis^23^). Without a single, consistent product, application studies may need to develop merging techniques, which may reveal (or add) biases across precipitation products that manifest as temporal discontinuities in derived model outputs, impairing detection of hydrometeorological phenomena such as drought^24,25^.

Estimating precipitation at ungauged locations is complicated by topographic features that drive orographic precipitation^26–28^. Water resources in the western U.S., Canada, and Mexico depend critically on mountain precipitation^29,30^. Yet most precipitation measurements are made at stations located at lower elevations. Naively interpolating values between lower-elevation stations across the intervening topography would systematically misrepresent the true precipitation field. Adding an orographic adjustment greatly improves the quality of precipitation estimates subject to topographic influences^31^.

The dataset presented in this article was developed for use in climate downscaling applications for the U.S. Bureau of Reclamation, although applications to drive land models and direct analyses are also anticipated. Relative to its methodological predecessors, this data set (herein L15) extends the domain of Livneh *et al.* (2013 (ref. 12): herein L13) into Mexico and Canada, and is both a slightly larger domain (i.e. all of Mexico south of 25° N) and finer spatial resolution (1/16° versus 1/8°) than Maurer *et al.* (2002 (ref. 32): herein M02), and incorporates a topographic adjustment over the entire domain unlike existing Mexico-only products^33,^ ^9^. This article discusses: (i) the newly incorporated (Mexican) station data and quality control procedures applied; (ii) implications of topographic adjustments and selection of the climate normal period on precipitation and hydrology, as represented by a hydrologically-based land surface model; (iii) an examination of spatio-temporal discontinuities relative to a reanalysis product over the North American Monsoon (NAM) region.

## Methods

### Station data and gridding process

**Figure 1:**
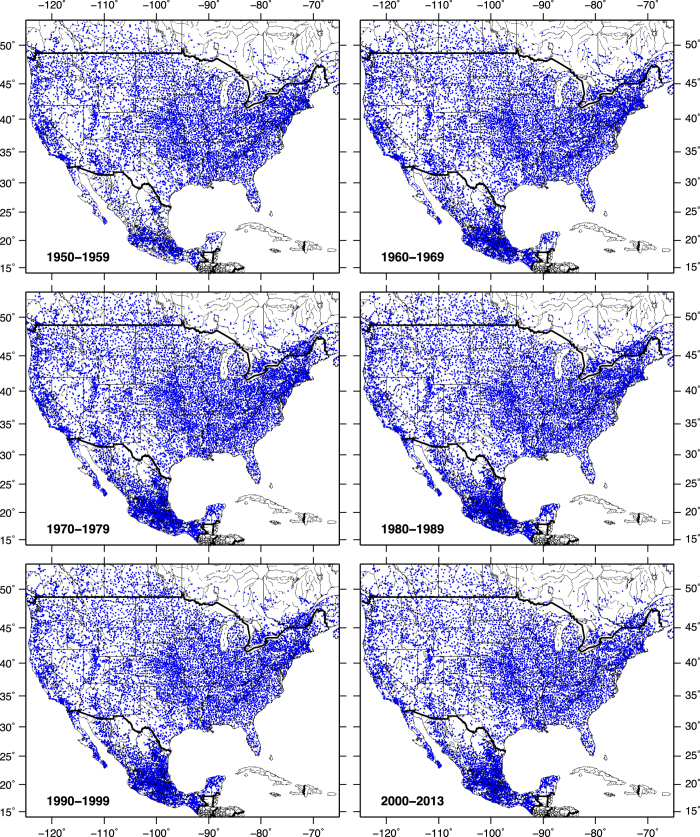
Station density illustration. Blue dots show stations included in the gridding process, by decade.

As described in L13, the gridding procedure used the SYMAP algorithm^34^ to follow the methodology originally used by M02. As in L13 (and M02), the stability constraint requiring a minimum of 20 years of data was applied only to CONUS and Canadian stations. In contrast, given the relative paucity of station data in Mexico, those data were screened following the procedure put forth in a previous gridded Mexican data set^33^ requiring a minimum of 50 valid days of data in any given year for a station to be included. Maps of decadal station density illustrate the temporal evolution of data availability (Fig. 1). Areas of low station density include central and eastern Canada, the U.S. southwest, and northern Mexico.

### Orographic scaling

Consistent with L13, two adjustments were made to the gridded meteorological fields to account for the effects of topography. First, temperatures were lapsed with elevation at a constant rate of 6.5 °C/km. Second, precipitation was scaled based on existing estimates of monthly climatological precipitation that were computed taking topographical effects into account. We used PRISM for this purpose within the CONUS, and the Vose *et al.* ^35^ extension to an older climatology^36^ elsewhere (herein V14). The key steps in precipitation scaling were therefore:

1. Starting with the SYMAP-interpolated data, compute the monthly mean precipitation at every point over a 30-year period (1981–2010, coincident with PRISM and V14 climate normals);
2. For each of the 12 months and for each point, compute the ratio between the topographically-aware PRISM or V14 climatological value and the climatological value from the interpolated data. Both PRISM and V14 (1/20° native resolution) were conservatively remapped to the 1/16° gridded resolution prior to taking ratios;
3. Multiply all daily precipitation values at that point and in that month by the ratio computed in step 2. This is done for the entire data record (1950–2013).

Features unique to this analysis relative to L13 were (i) orographic adjustments to locations outside CONUS, and (ii) the climatological reference period chosen for scaling within CONUS.

The older climatology^36^ for North America was updated following the methods of V14. Climate normals were computed for each station for the 1981–2010 base period. The irregularly spaced station normals were then interpolated to a 1/20° grid using trivariate thin-plate smoothing splines with latitude, longitude, and elevation as predictors^37^. Thin-plate splines are well suited for a large domain such as North America because the relationship between the dependent and predictor variables can vary in space, which facilitates the reconstruction of complex geographical patterns^38^.

Since the V14 reference period is 1981–2010, the same PRISM normal period was chosen for CONUS for transboundary consistency. This is in contrast to the 1961–1990 period used in L13 (and M02). The selected PRISM period overlaps with the monitoring era (post-1979) of critical precipitation observations at high elevations by the National Resource Conservation Service SNOw TELemetry (SNOTEL; where temperatures were not used).

### Quality control

**Figure 2:**
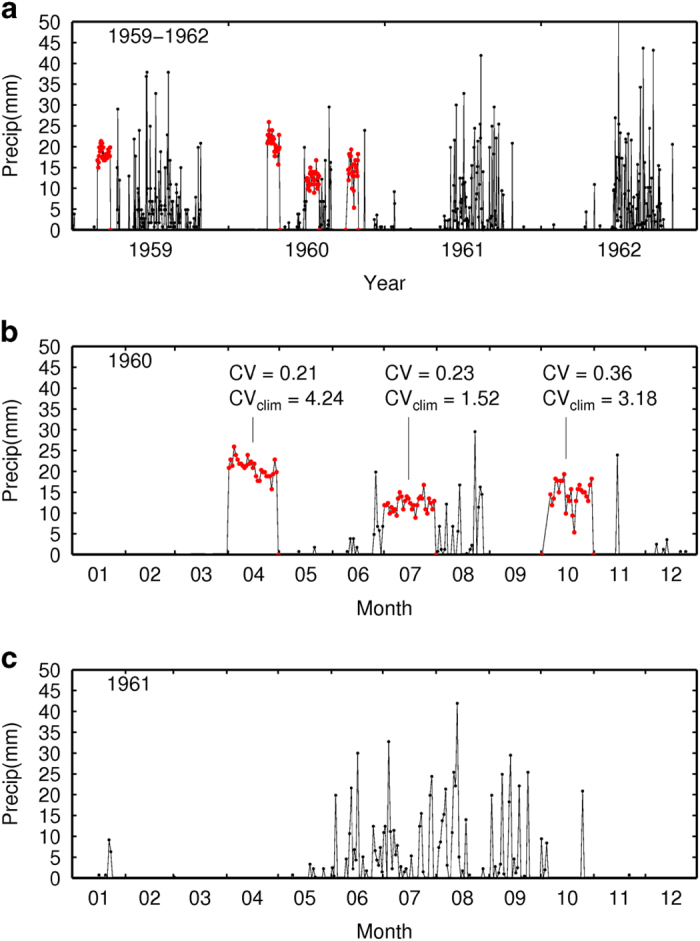
Demonstration of precipitation station quality control for Mexico. Illustration of spurious precipitation data (red circles) identified during a quality control procedure for Mexican station data—shown for Station ID: 14111, Poncitlan, Jalisco, Mexico. Values of the coefficient of variation (CV) of daily values for each of the months flagged as bad data in 1960 are listed, along with the climatological average CV values for those months. Panel (**a**) illustrates spurious records alongside records deemed non-spurious for a 4 year period, with respective panels (**b**) and (**c**) illustrating these features in greater detail for an individual year.

Descriptions here are largely restricted to the Mexican data and issues beyond those previously addressed by L13 for CONUS and Canadian stations. The first issue involved spurious precipitation data within the Mexican station record, with periods of near-constant non-zero precipitation, an example shown in Fig. 2. Erroneous values were identified and flagged on a monthly basis for each station by computing the monthly coefficient of variation, CV_i_ (the ratio of the standard deviation of daily values to their mean for month *i*) over the entire period of record; and its climatological average value, CV_m_ (*m*=1 to 12); and removing months with CV_i_<0.18 CV_m_, which was determined empirically from a training set of 25 station records, each longer than 15 years, taken from 7 states. In all cases examined, months flagged as spurious were clear outliers relative to the distribution of all CV values for the given month, with CV_i_ values that fell more than two standard deviations away from the mean CV for that month, CV_m_. We recognize that this procedure cannot detect spurious data that have similar CV values to real data; therefore this method should be considered conservative. By removing spurious months of data, we note that the gridding algorithm will subsequently search for another proximal station to estimate gridded precipitation, which may increase or decrease the estimate. Similarly, adding short-lived stations or subnetworks, as is particularly common for Mexico (short-term precipitation network described below), will affect the heterogeneity of the precipitation field, which we do not explicitly quantify here.

Another issue that arose during the gridding process involved the time of observation, TOBS, for Canadian stations, which were initially interpreted as local time readings (as is the case for CONUS) in L13. However, through a lagged correlation analysis it was determined that times were recorded in Greenwich Mean Time (GMT) resulting in an asynchronous offset in meteorological events, which was corrected by adjusting the station TOBS entries to local time.

### Code availability

Customized C++ code was used for the major gridding operation in this data set that is publically available alongside the data set at the Lawrence Livermore National Laboratory (LLNL) accessible here (ftp://192.12.137.7/pub/dcp/archive/OBS/livneh2014.1_16deg/). Further, processing of the data in network Common Data Form (netCDF) format was done for remapping, aggregating, and scaling of climatologies using open source Climate Data Operators (CDO) and netCDF Operator (NCO) utilities. Hydrologic simulations were performed with publicly available model code (http://www.hydro.washington.edu/Lettenmaier/Models/VIC/).

## Data Records

A list of data sources used to build this data set are included in Table 1. Daily Canadian precipitation, maximum and minimum temperature station data were obtained from Environment Canada, and CONUS data from the National Climatic Data Center (NCDC^39^). For Mexico data were provided by the Servicio Meteorológico Nacional (SMN), under the Comisión Nacional del Agua (CONAGUA), for the period 1950–2013. However, since 2000, a sharp decline in Mexican station density was noted in several states, particularly after 2006. To fill gaps for the period 2000–2013, we contacted CONAGUA’s regional offices in the states of Chihuahua, Sonora, Coahuila, Durango, Nayarit Puebla, and Yucatan. Additionally, 86 precipitation stations distributed across the Sierra Madre Occidental from the North American Monsoon Experiment Event Rain Gauge Network (NERN)^40^ were incorporated, which provide an important sampling of high elevation precipitation absent from the above station sources.

The final data set contains gridded station data for precipitation, daily maximum and minimum temperature, in addition to wind^41^ data from National Centers for Environmental Preciction (NCEP) National Centers for Atmospheric Research (NCAR) reanalysis. Also included are hydrologic model outputs over Mexico, CONUS, and southern Canada at a 1/16° resolution for the period 1950–2013. The data are intended to support downscaling and long-term hydroclimatic studies. The meteorological data are archived at the National Oceanic and Atmospheric Administration (NOAA) National Centers for Environmental Information (NCEI), with access details provided in the Data Citation 1. An additional copy of the data, including derived hydrologic output are hosted in a public repository, the LLNL site listed above. Variables (Table 2) are provided in netCDF format.

## Technical Validation

**Figure 3:**
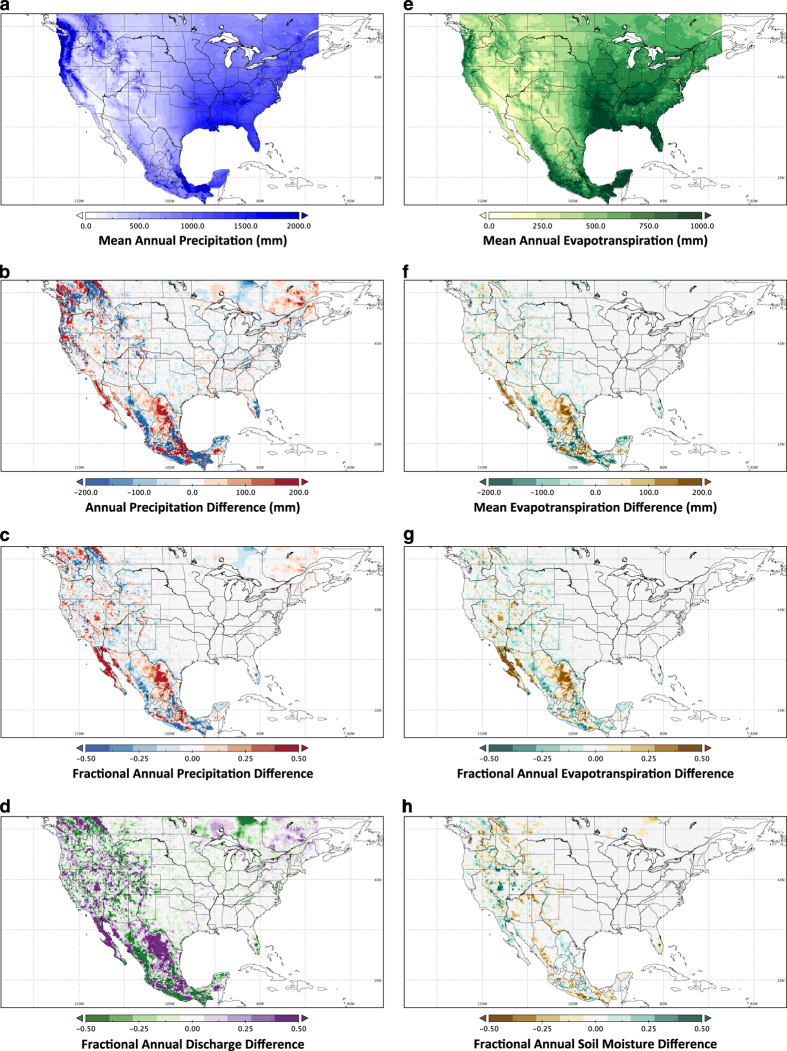
Orographic scaling comparisons. Differences in mean annual conditions (1950–2013) between two precipitation scaling scenarios: the first is similar to L13 (i.e., the CONUS scaled to PRISM 1961–1990 normals and no scaling elsewhere), while the second case is the present data set (scaled to PRISM 1981–2010 within CONUS and Vose *et al.*^35^ elsewhere). Differences show the former case minus the latter. (**a**) shows precipitation for the present case, (**b**) and (**c**) show precipitation differences, (**e**) shows ET simulated by the VIC hydrologic model using the present precipitation scaling case; differences between the two scaling scenarios in discharge (Q=runoff+baseflow) are shown in (**d**), evapotranspiration (**f**,**g**) and soil moisture (**h**).

The impact of scaling CONUS precipitation to PRISM climate normals over the period 1981–2010 rather than 1961–1990 (as used in L13 and M02) is illustrated in Fig. 3. Scaling ratios were computed separately for the corresponding 30-year periods for the gridded data relative to the respective normals. Outside CONUS, Fig. 3 shows the difference between scaling precipitation to V14 and using no scaling.

To understand potential hydrologic impacts of the scaling choice, the Variable Infiltration Capacity (VIC) hydrologic model^42^ was run with the meteorological forcing data developed here, as well as with required wind data that were interpolated through a bi-cubic approach from a larger (approximately 1.9° grid) reanalysis grid^41^. VIC model parameters in CONUS were obtained from L13, who validated model discharges over major CONUS river basins. 1/16° parameters over Mexico were obtained from previously published work^9^, for which discharges were validated in selected basins^7,33^. Canadian parameters were obtained at 1/8° from M02 and disaggregated to 1/16° using a nearest neighbor approach. We advise users of the simulated hydrologic states and fluxes to judge the fidelity of those simulations by referring to the aforementioned analyses that calibrated and validated VIC over most of CONUS and parts of Canada and Mexico, respectively.

Outside CONUS, precipitation differences between the scaled (V14 1981–2010) and unscaled grids can be substantial, up to 400 mm/yr in mountainous regions, which translate to fractional differences up to 75% although typically less than 25% over these regions. Particularly large differences are found in the Canadian Rocky Mountains and Coastal Range, the Sierra Madre (Occidental and Oriental), and the Baja Peninsula—areas of enhanced topographical relief—with progressively smaller differences over the modest topography of eastern Canada and flatter areas.

Within CONUS, precipitation was scaled to PRISM 1981–2010 for consistency with Mexico and Canada. Differences between this scaling and the 1961–1990 found in L13 and M02 are still notable, although generally less than 200 mm/yr with the exception of wet coastal areas, which altogether translate to fractional differences of less than 50% and typically less than 25%. For all areas, fractional differences in simulated total discharge between the two scaling cases are of slightly larger fractional sensitivity than precipitation, and are relevant for water resource management. Differences in ET and total column soil moisture are less sensitive than discharge, exhibiting sensitivities generally less than 25%/50% inside/outside CONUS between scaling cases.

### Spatio-temporal continuity, transboundary consistency, and the north American monsoon

**Figure 4:**
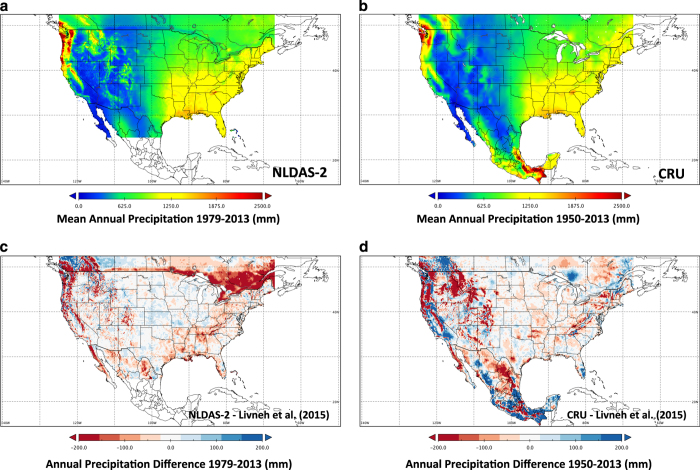
Inter-dataset precipitation comparison over the full domain. Mean annual precipitation (1979–2013) in (**a**) and NLDAS2 and (**b**) CRU v.3.22, as well difference maps NLDAS2 minus L15 (**c**) and CRU minus L15 (**d**). There is a notable transboundary discontinuity in NLDAS2, and NLDAS2 is generally drier than L15.

The L15 dataset offers several advantages over other gridded observational products that cross international boundaries (Fig. 4). To illustrate the advantages of L15, we compare it with the commonly used 1/8°-resolution North American Land Data Assimilation System phase-2 (NLDAS2) product (104 google scholar citations^21^) as well as the Climate Research Unit (CRU^43^) data (461 google scholar citations). Limitations of NLDAS2 relative to L15 include: (i) shorter duration (NLDAS2 begins in 1979); (ii) lack of coverage south of 25° N; and (iii) transboundary discontinuities in both the topographic correction (derived from PRISM over the US, but not performed over Canada or Mexico) and the source of the precipitation data used. Over CONUS, NLDAS2 uses NCDC gauge data. Over Canada, NLDAS2 uses the North American Regional Reanalysis (NARR/R-CDAS) product, disaggregated from its coarser 32 km resolution. Over Mexico, NLDAS2 uses a hierarchy of data sets, with the first choice being a 1/4° gauge product (1° resolution prior to 2001), temporally disaggregated based on CMORPH satellite-retrieved 8 km data; followed by other data sets when these are not available (for more details, see: http://ldas.gsfc.nasa.gov/nldas/NLDAS2forcing.php#AppendixC). The major limitation of CRU relative to L15 is its comparatively coarse spatial resolution (0.5°) and temporal resolution (monthly), while it offers the advantage of being globally available and suitable for trend analyses given its gridding algorithm that computes station-anomalies about a normal period.

Figure 4 compares mean annual precipitation between L15 and NLDAS2 for their common period, as well as L15 and the CRU data, v.3.22 for the full period (1950–2013). The CRU data do not suffer any spatial clear spatial discontinuity, however, they clearly exhibit a smoother surface (Fig. 4, panel b) relative to L15 (Fig. 3). The difference map (Fig. 4, panel d) generally suggests that CRU underestimates precipitation in regions of high precipitation (i.e. mountainous terrain) and slightly underestimates in low precipitation regions relative to L15, which might be expected given its coarser resolution. The two exceptions are on the Yucatan Peninsula (MX) and the Canadian Rocky Mountains, where CRU overestimates precipitation relative to L15. Although the authors of CRU suggest ‘This dataset should only be used for climate trend analysis, …’^43^, we feel the comparisons are worthwhile to highlight major differences with that widely used dataset.

Both the raw NLDAS2 (Fig. 4, panel a) and the difference field (Fig. 4, panel c) show a clear discontinuity at the Canadian border, as well as another discontinuity at 50° N, indicating substantial dry biases in NLDAS2 over the Canadian portions of the Columbia and St Lawrence basins. A less dramatic discontinuity at the US-Mexico border leads to similar dry biases in NLDAS2 over the Mexican portions of the Colorado River and Rio Grande/Rio Bravo basins. Overall, L15 is generally wetter than NLDAS2 over areas of topographical complexity.

Discontinuities at the US-Mexico border are particularly important to studies of the North American Monsoon. Summer monsoon rains provide 30–80% of the annual precipitation across northwestern Mexico and the southwestern US between 20° and 35° N (ref. 44). Predicting and understanding this highly variable regional water resource is vital for planning efforts, yet models have difficulty reproducing monsoon precipitation, leading to poor forecast skill^45^.

**Figure 5:**
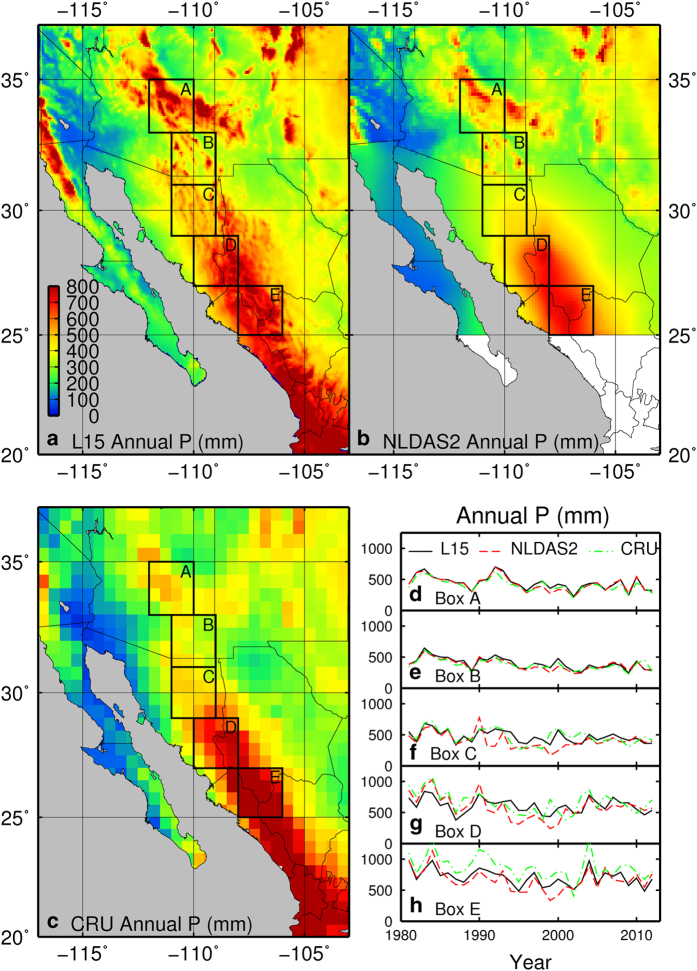
Inter-dataset precipitation comparison for the North American Monsoon region. Annual total precipitation over the period 1981–2012, showing (**a**–**c**) maps of mean annual total precipitation from L15, NLDAS2, and CRU, respectively; and (**d**–**h**) timeseries of spatial average annual total precipitation from all products for the five boxes labelled A-E in (**a**–**c**).

L15 represents a substantial improvement over existing datasets in the monsoon region. As shown in Fig. 5a–c, L15 and CRU completely cover the monsoon region, but NLDAS2 ends at 25° N. Between 25° N and the US-Mexico border, the lack of an elevation correction in NLDAS2 leads to a precipitation field that has not only a diffuse spatial distribution and unrealistic relationship to topography, but also a substantial discontinuity at the US-Mexico border. L15 and CRU give summer precipitation totals that are more consistent with previous observational studies^46^. However, CRU’s relatively coarse spatial resolution (0.5°) limits its usefulness in basin-scale hydrologic studies within the North American Monson region, due to the topographic complexity there. It is worth noting that other available products not shown here suffer from similar problems to those shown: the North American Regional Reanalysis (NARR^47^) lacks an elevation correction over Mexico, leading to a discontinuity similar to that of NLDAS2; other global gridded meteorology datasets such as the Global Precipitation Climatology Project (GPCP^48^) have coarser spatial resolution than CRU.

Temporal differences among these products also exhibit a discontinuity at the US-Mexico border. Along a transect of 2×2° boxes from central Arizona to southern Sonora (boxes A-E in Fig. 5a–c), the timeseries of annual precipitation from the three products over the period 1981–2012 agree remarkably in the US (boxes A and B; Fig. 5d,e), but differ noticeably over Mexico, particularly during the 1990s (boxes C-E; Fig. 5f–h). All three products capture the 1995–2004 drought^49,50^ in the Yaqui River basin (boxes B-D) to varying extents (Fig. 5e–g). L15 bears the strongest resemblance to a previously published timeseries for the region^49^ using 97 meteorological stations in Arizona and Sonora, including relatively high precipitation in years 1991–1994, followed by a declining trend through 2004, punctuated by above average precipitation in 1997 and 2000. However, in boxes C and D (Fig. 5d,e), NLDAS2 begins the drought early (in 1991) and CRU ends the drought early (in 2000 in box C and in 2004 in box D). Thus, while differences among precipitation products warrant further investigation, the L15 dataset promises to be of major benefit to studies of hydrology and water resources in the North American Monsoon region.

## Usage Notes

We caution users that similar to predecessor data sets, M02 and L13, the L15 data presented here are not suitable for trend analysis, since they use many stations that do not span the full temporal period 1950–2013. Hence, sampling evolves over time, especially for the country of Mexico over which we used a much less restrictive stability constraint, i.e., >50 days of valid data required for station inclusion, versus >20 years of data for CONUS and Canadian stations. Despite our best efforts to minimize transboundary discontinuities, we identified a discontinuity in daily minimum temperature, Tmin, across the MX/US. In fact, no discontinuities are apparent in seasonal climatologies for Tmax, Tmin, or precipitation, yet when exploring a trend analysis, we note a small downward trend in Tmin in central and northern Mexico, that is opposite in sign north of the US/MX border. We investigated other data sets (not shown) and found a similar discontinuity in trend in the CRU data v.3.0, which is greatly reduced in versions v.3.10 and later (the most recent version is v.3.22 as of 15 July, 2015). The more recent CRU versions switched from gridding Tmax and Tmin separately, in favour of gridding Tmean and diurnal temperature range (DTR) from which Tmax and Tmin are inferred. We further investigated this issue internally and note a downward trend in station elevations over much of Mexico, suggestive of increased sampling of inversion processes causing the downward trend in Tmin over time.

## Additional Information

**How to cite this article:** Livneh, B. *et al.* A spatially comprehensive, hydrometeorological data set for Mexico, the U.S., and Southern Canada 1950–2013. *Sci. Data* 2:150042 doi: 10.1038/sdata.2015.42 (2015).

## Supplementary Material



## Figures and Tables

**Table 1:** Summary of daily data sources used to build the present data set

**Data type**	**Source**	**Details**
Precipitation, Maximum and Minimum Temperature	CONUS: NCDC (ftp://ftp.ncdc.noaa.gov/pub/data/ghcn/daily/ ^38^)	Precipitation CONUS: 21137 total stations; 8844 satisfy stability constraintCanada: 7967 total station; 2245 satisfy stability constraintMexico 5430 stations all satisfy the selection criteria (86 stations from NERN)
	Canada: Environment Canada	Maximum Temperature CONUS: 16919 total stations; 6159 satisfy stability constraintCanada: 7841 total station; 1875 satisfy stability constraintMexico 5246 total stations; 5220 satisfy the selection criteria
	Mexico: Servicio Meteorológico Nacional, under the Comisión Nacional del Agua* † ‡ §	Minimum Temperature CONUS: 16983 total stations; 6275 satisfy stability constraintCanada: 7967 total station; 1913 satisfy stability constraintMexico 5243 total stations; 5220 satisfy the selection criteria
Wind data	NCEP–NCAR reanalysis^41^	
The decadal average and standard deviation of the number of stations reporting for each country within the sampling domain is Canada: 1861, 362; CONUS: 7489,476; Mexico: 2611, 772, respectively		

*Puebla, Boris Isauro Hernandez, Jefe del Centro de Prevision Meteorologica. Email: boris.hernandez@conagua.gob.mx phone: 222-211-8378

^†^ Tlaxcala, Juana Angelica Diaz Trenado, Jefe del Departamento de Hidrometria. Email: juana.diaz@conagua.gob.mx phone: 246-468-9305

^‡^ Nayarit, Ruben Cambero Borrayo, Residente del area de infraestructura hidroagricola, Email: ruben.cambero@conagua.gob.mx phone: 311-214-2385

^§^ Nayarit, Arnulfo Saldierna, Asistencia tecnica y control de calidad, Email: arnulfo.saldierna@conagua.gob.mx phone: 311-210-4346 (director's office).

**Table 2:** List of publically available daily hydrometeorological variables.

**Variable Name**	**Units**	**Source**
Precipitation	mm	station
Maximum Temperature	°C	station
Minimum Temperature	°C	station
Wind speed	m/s	reanalysis
Surface Runoff	mm	VIC
Baseflow	mm	VIC
Total ET	mm	VIC
Snow Water Equivalent	mm	VIC
Soil Moisture	mm	VIC
Canopy Moisture	mm	VIC
Latent Heat Flux	W/m^2^	VIC
Sensible Heat Flux	W/m^2^	VIC
Ground Heat Flux	W/m^2^	VIC
Net Radiation	W/m^2^	VIC
Potential ET	mm	VIC
